# Genetic polymorphisms in *CYP1A1*, *GSTM1*, *GSTP1* and *GSTT1* metabolic genes and risk of lung cancer in Asturias

**DOI:** 10.1186/1471-2407-12-433

**Published:** 2012-09-27

**Authors:** M Felicitas López-Cima, Sara M Álvarez-Avellón, Teresa Pascual, Ana Fernández-Somoano, Adonina Tardón

**Affiliations:** 1 Molecular Epidemiology of Cancer Unit, University Institute of Oncology, University of Oviedo, C/Fernando Bongera, s/n, Oviedo, 33006, Spain; 2 CIBER en Epidemiología y Salud Pública (CIBERESP), Institute of Health Carlos III, C/Melchor Fernández Almagro, 3-5. Pabellón 9, planta baja, 28029, Madrid, Spain; 3 Pneumology Department, Cabueñes Hospital, Cabueñes, s/n, Gijón, 33394, Spain

**Keywords:** Lung cancer, Polymorphisms, Metabolic genes, CYP1A1, GSTM1, GSTP1, GSTT1

## Abstract

**Background:**

Metabolic genes have been associated with the function of metabolizing and detoxifying environmental carcinogens. Polymorphisms present in these genes could lead to changes in their metabolizing and detoxifying ability and thus may contribute to individual susceptibility to different types of cancer. We investigated if the individual and/or combined modifying effects of the *CYP1A1 MspI* T6235C, *GSTM1 present/null*, *GSTT1 present/null* and *GSTP1 Ile105Val* polymorphisms are related to the risk of developing lung cancer in relation to tobacco consumption and occupation in Asturias, Northern Spain.

**Methods:**

A hospital-based case–control study (CAPUA Study) was designed including 789 lung cancer patients and 789 control subjects matched in ethnicity, age, sex, and hospital. Genotypes were determined by PCR or PCR-RFLP. Individual and combination effects were analysed using an unconditional logistic regression adjusting for age, pack-years, family history of any cancer and occupation.

**Results:**

No statistically significant main effects were observed for the carcinogen metabolism genes in relation to lung cancer risk. In addition, the analysis did not reveal any significant gene-gene, gene-tobacco smoking or gene-occupational exposure interactions relative to lung cancer susceptibility. Lastly, no significant gene-gene combination effects were observed.

**Conclusions:**

These results suggest that genetic polymorphisms in the *CYP1A1*, *GSTM1*, *GSTT1* and *GSTP1* metabolic genes were not significantly associated with lung cancer risk in the current study. The results of the analysis of gene-gene interactions of *CYP1A1 Msp*I T6235C, *GSTM1* present/null, *GSTT1* present/null and *GSTP1* Ile105Val polymorphisms in lung cancer risk indicate that these genes do not interact in lung cancer development.

## Background

Established risk factors for lung cancer include exposure to cigarette- and environmental-derived pro-carcinogens. Cigarette smoking accounts for 80% to 90% of cases among men and 55% to 80% of cases among women [1]. Occupational exposures in industrial facilities account for an additional 9% to 15% of lung cancer cases [2]. However, although cigarette smoking and occupation are the major causes of lung cancer, only a small fraction of smokers and workers in high-risk occupations develop this disease. This suggests other causes, including genetic susceptibility, may contribute to the variation in individual lung cancer risk. This genetic susceptibility may partially result from inherited polymorphisms in the genes involved in carcinogen metabolism [3-5]. Thus, many toxic compounds implicated in carcinogenesis require both activation by metabolic enzymes classified as Phase I and detoxification by enzymes classified as Phase II. Genetic changes in genes that encode metabolic Phase I enzymes and detoxification Phase II enzymes are linked to increases in metabolic activation and decreases in metabolic detoxification of environmentally derived pro-carcinogens and may increase lung cancer susceptibility.

Phase I enzymes (e.g., CYP) oxidize a wide range of substrates, resulting in metabolically active carcinogens. For instance, *CYP1A1* is responsible for the metabolic activation of polycyclic aromatic hydrocarbons (e.g., benzo[a]pyrene), a leading pro-carcinogen found in cigarette smoke and environmental pollution [6]. In addition, the *CYP1A1 Msp*I polymorphism in the 3’-flanking region of the *CYP1A1* gene [7] is in strong linkage disequilibrium with a non-synonymous SNP of an isoleucine to valine amino acid change at codon 462 [8]. Studies suggest that these 2 *CYP1A1* SNPs are implicated in lung cancer risk [9-11].

Phase II enzymes (e.g., the GST supergene family) play a central role in the detoxification of toxic and carcinogenic electrophilic compounds. GSTs are a large family of cytosolic enzymes that catalyze the detoxification of potential carcinogens through a conjugation with reduced glutathione. GSTM1 and GSTP1 metabolize large hydrophobic electrophiles, such as polycyclic aromatic hydrocarbon-derived epoxides [12]. GSTT1, on the other hand, is involved in the metabolism of smaller compounds, such as monohalomethane and ethylene oxide [13]. GSTs also metabolize compounds formed during oxidative stress, such as hydroperoxides and oxidized lipids, and they are transcriptionally activated during oxidative stress [14].

Certain genetic variants in the glutathione S-transferase genes, such as the *GSTM1* and *GSTT1* null polymorphisms, are prevalent among 50% and 20% of Caucasians, respectively [15], result in the lack of active enzyme [16]. Meta-analyses have indicated that the carriers of *GSTM1* null or *GSTT1* null genotypes have a slightly higher risk of developing lung cancer compared to carriers of at least one functional allele [17-19]. GSTP1 is the major isoenzyme expressed in human lung tissue [20]. A A/G single nucleotide polymorphism (SNP) located within the substrate-binding domain of the *GSTP1* results in an isoleucine to valine amino acid change at codon 105 (Ile105Val). Notably, the valine allele is associated with a lower conjugating activity when compared to the isoleucine allele [21-23]. The frequency distribution of the *GSTP1* Val allele varies across racial/ethnic groups [20]. However, epidemiological studies of the impact of the *GSTP1* Ile105Val polymorphism on lung cancer risk, including two meta-analyses, show inconsistent results [19,24-27].

Many studies investigating the association between the *CYP1A1 Msp*I T6235C, *GSTM1 present/null*, *GSTT1 present/null*, and *GSTP1 Ile105Val* polymorphisms and lung cancer risk have been limited by small sample sizes, leading to a lack of statistical power [28-31]. Furthermore, pooled analyses to increase sample size have led to conflicting results between groups, most likely due to population differences (i.e., ethnicity) or failure to control for other potential confounders, including age and sex [32]. Therefore, the four genes analysed in this study encode enzymes involved in the metabolism of polycyclic aromatic hydrocarbons (PAHs) and aromatic amines, which are procarcinogens present in both smoking and occupation, and thus, both variables must be controlled for in the analysis. This study will show an analysis of occupation as a method to verify whether individuals who possess at least one variant allele of the polymorphisms studied and belong to list A occupation have a higher risk of lung cancer than those individuals with the wild-type genotype.

To examine whether genetic polymorphisms in Phase I and Phase II metabolic genes are associated with lung cancer risk, we studied 4 polymorphisms in the *CYP1A1*, *GSTM1*, *GSTT1* and *GSTP1* metabolic genes, individually and combined, in a large hospital-based case–control study of lung cancer including 789 lung cancer cases and 789 controls from a Caucasian population in Asturias, Northern Spain. Moreover, we analyzed the possible interactions gene-tobacco and gene-occupational exposure.

## Methods

### Study population

The CAPUA (Lung Cancer in Asturias [Cáncer de Pulmón en Asturias], Spain) study is a hospital-based, case–control study conducted by the Molecular Epidemiology Cancer Unit at the University Institute of Oncology (University of Oviedo). Details of the study design and methods have been described elsewhere [33-37]. Briefly, from October 2000 to December 2010, a standard protocol was used to recruit incident cases of histologically confirmed lung cancer at Asturias’ four main hospitals (the Cabueñes Hospital in Gijón, San Agustin Hospital in Avilés, General Hospital in Oviedo and Álvarez-Buylla Hospital in Mieres). In addition, controls were selected from patients admitted to those hospitals with diagnoses unrelated to the exposures of interest and individually matched by ethnicity, gender, age (± 5 years) and hospital. The main specific pathologies of the final controls selected were as follows: 36.6% inguinal and abdominal hernias (ICD-9: 550–553), 29.3% injuries (ICD-9: 800–848, 860–869, 880–897), and 12.5% intestinal obstructions (ICD-9: 560, 569, 574). The CAPUA study was approved by the respective ethics committees of the hospitals involved, and written consent was obtained from all participants.

### Data collection

During the first hospital admission, information on known or potential risk factors for lung cancer was collected personally by trained interviewers using computer-assisted questionnaires. These structured questionnaires collected data from each participant on age, gender, socio-demographic characteristics, recent and past tobacco use, personal and family history of lung cancer, and occupational history.

Participants were categorized by smoking status into three groups: non-smokers, defined as subjects who had not smoked at least one cigarette per day regularly for six months or longer in their lifetimes; former smokers that included regular smokers who had stopped smoking at least five years before the interview; and current smokers who met none of the previous criteria. Smoking intensity (pack-years (PY)) was defined as the number of packs of cigarettes smoked per day multiplied by the number of years of smoking. Subjects were also categorized as light (<37 PY) or heavy (≥37 PY) smokers, based on the mean cumulative tobacco consumption in the control group.

For each job held for a minimum of 6 months or longer, we obtained information on the industry name, production type, job title, and the year in which the job began and ended. Occupations and industries were coded using the 1977 Standard Occupational Classification [38] and 1972 Standard Industrial Classification schemes [39]. Lastly, each coded occupation was categorized according to the list of occupations known to be associated with lung cancer (List A) based on evaluations of carcinogenic risks by the International Agency for Research on Cancer (IARC) [40,41]. This list is periodically updated and has been extensively used worldwide as a standardized tool to quantify the burden of occupational lung cancer [42-47]. Some examples of List A occupations among our individuals are the following: Arsenic, uranium, iron-ore, asbestos and talc miners; Ceramic and pottery workers; Iron and steel founding (casters, moulders and core makers); Copper, zinc, cadmium, aluminum, nickel chromates, beryllium blue collar workers; Platters; Shipyard/dockyard, railroad manufacture workers; Coke plant and gas production workers; Insulators, roofers and asphalt workers; and painters.

### Genotype analysis

Laboratory personnel were blinded to case and control status. Genomic DNA was extracted from peripheral blood samples (97.6% of total) or exfoliated buccal cells (2.4% of total) as previously described [48]. As quality control steps, genotyping was repeated randomly in at least 5% of the samples, and two of the authors independently reviewed all results. In this quality control there was 100% concordance between the replicate samples and genotype calls between the independent evaluator. The null genotype of *GSTM1* and *GSTT1* was determined by multiplex polymerase chain reaction (PCR) using β-globin as an internal positive control and previously described primers and conditions [49]. The polymorphisms in *CYP1A1* and *GSTP1* (rs1695) were analysed by polymerase chain reaction (PCR) combined with restriction fragment length polymorphism (RFLP) using previously described primers and conditions [50,51]. PCR was performed in a 10 μl mixture containing 20 ng of genomic DNA, 0.25 mM of each dNTP, 0.5 units of *Taq* polymerase (Biotools), and 10 pmol of each primer in a 1x PCR buffer. PCR products were digested overnight with the indicated restriction enzyme at 37°C. DNA fragments were resolved on agarose gels and stained with ethidium bromide. To verify that the data obtained by RFLP coincided with the allele sequence, representative fragments were further purified for PCR-directed sequencing to confirm the different polymorphisms (data not shown).

### Statistical analysis

Statistically significant departures from Hardy-Weinberg equilibrium were evaluated by comparing observed and expected genotype frequencies among controls using a chi-square test with 2 degrees of freedom. Differences in the distribution of categorical data (gender, smoking status, family history of lung cancer, and occupational status) were tested using a chi-square test. Continuous variables that were not normally distributed among controls (age, PY) were assessed using a non-parametric Mann–Whitney U test. Crude odd ratios (ORs) were calculated using Wolf's method [52]. Multivariate unconditional logistic regression analysis with adjustment for age, family history of any cancer, tobacco consumption and worker in list A occupation (no, yes) was performed to calculate adjusted ORs and 95% confidence intervals (CIs). Gene-gene and gene-environment interactions were estimated using a logistic regression model, which included an interaction term as well as variables for exposure (tobacco consumption, family history of any cancer, and worker in list A occupation), genotypes (*CYP1A1*, *GSTM1*, *GSTT1* or *GSTP1*) and potential confounders (age).

To analyze the gene-gene interactions, the genotypes of two genes were combined and sorted into four categories consisting of no risk alleles (the reference group), no risk allele for the first gene and any risk allele for the second, any risk allele for the first gene and no risk allele for the second, and two risk alleles. For C*YP1A1* the C-allele was classified as the putative high risk allele. In the case of GSTs genes, the putative high-risk alleles were the ≥1 null allele for *GSTM1*, the ≥1 null allele for *GSTT1* and, finally, the Val allele for *GSTP1*.

The sample size of our study for an allele frequency between 11–35% is sufficient to detect ORs greater than 1.34 or lower than 0.69 with more than 80% power assuming a dominant genetic model.

All statistical analyses were performed using STATA 8.0 software (Stata Corporation, College Station, Texas).

## Results

### Subject characteristics

The analysis included 789 lung cancer cases and 789 controls from a Caucasian population of Asturias, Northern Spain (CAPUA Study, acronym for *CÁncer de PUlmón enAsturias* [Lung Cancer in Asturias]). There were no statistically significant differences among the cases and controls regarding gender. There were statistically significant differences comparing the cases to controls regarding median age (67 vs. 66), tobacco smoking pack-years (PY) (54 vs. 30.1), family history of lung cancer (11.4% vs. 6.5%) and list A occupation status (List A include occupations known to be associated with lung cancer) (8.8% vs. 13.2%). There were more current smokers (63.7% vs. 34.3%) and more heavy smokers (62.29 vs. 36.89 PY) among the cases than among the controls. Histologically, squamous cell carcinoma (39.8%) and adenocarcinoma (31.3%) were the main types of lung cancer (summarized in Table 1).

**Table 1:** **Characteristics of lung cancercases and controls**

**Characteristic**	**Cases (n = 789)**	**Controls (n = 789)**	**p**^**a**^
	**n (%)**	**n (%)**	
Gender			
Male	697 (88.3)	697 (88.3)	1.000
Female	92 (11.7)	92 (11.7)	
Age (yrs), median (range)	67 (33–84)	66 (30–87)	0.034
n	789	789	
Smoking Status			
Never	51 (6.5)	222 (28.1)	<0.001
Ever	738 (93.5)	567 (71.9)	
Former	231 (29.8)	291 (37.3)	<0.001
Current	494 (63.7)	268 (34.3)	
Pack-years ^b^, median (range)	54 (0.65-274.5)	30.1 (0.05-170)	<0.001
n	736	559	
Family history of any cancer			
None	431 (57.2)	467 (59.9)	.004
Other cancers	237 (31.4)	262 (33.6)	
Lung cancer	86 (11.4)	51 (6.5)	0
Worker in list A occupation^c^			
No	641 (81.2)	685 (86.8)	0.002
Yes	148 (18.8)	104 (13.2)	
Histological type			
Squamous cell carcinoma	313 (39.8)		
Adenocarcinoma	246 (31.3)		
Small cell carcinoma	133 (16.9)		
Large cell carcinoma	24 (3.1)		
Non-differentiated	49 (6.2)		
Others	9 (1.1)		
Clinical diagnosis	13 (1.6)		
Missing	2		

^a^ Two-sided Chi-squared test and Mann–Whitney test where appropriate.

^b^ Pack-years for ever smokers.

^c^ List A includes occupations known to be associated with lung cancer.

We evaluated the impact of polymorphisms detected in 4 in Phase I and Phase II metabolism genes (*CYP1A1 MspI* T6235C, *GSTM1 present/null*, *GSTT1 present/null* and *GSTP1 Ile105Val*) on the risk of developing lung cancer. Within our study set, in heritance of at least one GST (M1, T1) deletion or GSTP1 105Val alleles were fairly common among controls with frequencies ranging from 21.3-58.1%, as detailed in Table 2. The genotype frequencies were comparable to other many European populations. The genotype frequencies did not substantially deviate from expected distribution under the Hardy-Weinberg Equilibrium (p ≥ 0.05).

**Table 2:** **Analysis of polymorphisms andlung cancer risk**

**Metabolic enzyme Gen SNP**	**Genotype**	**Cases**	**Controls**	**p**^**a**^	**Unadjusted OR**	**Adjusted**^**b**^**OR**
		**n (%)**	**n (%)**		**[95% CI]**	**[95% CI]**
**Phase I *CYP1A1****Msp*I T6235C	T/T	608 (77.1)	632 (80.1)	0.203	Reference	Reference
	T/C	172 (21.8)	145 (18.4)		1.23 [0.96-1.58]	1.16 [0.87-1.53]
	C/C	9 (1.1)	12 (1.5)		0.78 [0.33-1.86]	0.83 [0.31-2.20]
	T/C + C/C	181 (22.9)	157 (19.9)	0.141	1.20 [0.94-1.53]	1.13 [0.86-1.49]
**Phase II *GSTM1*** present/null	present/present	375 (48.3)	358 (46.1)	0.387	Reference	Reference
	> = 1 null allele	401 (51.7)	418 (53.9)		0.92 [0.75-1.12]	0.95 [0.76-1.19]
**Phase II *GSTT1*** present/null	present/present	618 (79.6)	611 (78.7)	0.662	Reference	Reference
	> = 1 null allele	158 (20.4)	165 (21.3)		0.95 [0.74-1.21]	0.85 [0.64-1.12]
**Phase II *GSTP1*** Ile105Val	Ile/Ile	352 (44.7)	330 (41.9)	0.391	Reference	Reference
	Ile/Val	339 (43.0)	366 (46.4)		0.87 [0.70-1.07]	0.84 [0.66-1.06]
	Val/Val	97 (12.3)	92 (11.7)		0.99 [0.72-1.36]	0.83 [0.57-1.19]
	Ile/Val + Val/Val	436 (55.3)	458 (58.1)	0.263	0.89 [0.73-1.09]	0.83 [0.66-1.05]

^a^ Chi-squared p-value.

^b^ Adjusted by age, pack-years (non-smoker, <37PY, ≥37PY), family history of any cancer, and worker in list A occupation^*^.

^*^ List A includes occupations known to be associated with lung cancer.

We evaluated the main effects of phase I/phase II xenobiotic metabolism genes (CYP1A1, GSTM1, GSTP1 and GSTT1) in relation to lung cancer susceptibility using univariate as well as multivariate statistics. No significant individual gene effects were observed among carriers of one or more CYP1A1 MspI 6235C (OR = 1.13; 95% CI = 0.86-1.49); GSTM1 null (OR = 0.95; 95% CI = 0.76-1.19); GSTT1 null (OR = 0.85; 95% CI = 0.64-1.12); GSTP1 Val (OR = 0.83; 95% CI = 0.66-1.05), as summarized in Table 2.

### Individual effects of CYP1A1 and GST SNPs on lung cancer and histological subtypes

No association was found between *CYP1A1 Msp*I T6235C polymorphism and lung cancer risk (adjusted OR = 1.16; 95% CI = 0.87-1.53; adjusted OR = 0.83; 95% CI = 0.31-2.20; adjusted OR = 1.13; 95% CI = 0.86-1.49 for T/C genotype, C/C genotype and T/C + C/C genotypes, respectively).

For the *GSTM1* present/null polymorphism, the frequency of the *GSTM1 null* genotype was lower in the cases (51.7%) than in the controls (53.9%), although not statistically significant. When we analyzed the association between the *GSTM1* genotypes and lung cancer risk, we found that the ≥1 null allele was no associated with the risk of developing lung cancer (adjusted OR = 0.95; 95% CI = 0.76-1.19).

In the case of the *GSTT1* present/null polymorphism the frequency of the *GSTT1 null* genotype was lower in the cases (20.4%) than in the controls (21.3%), although not statistically significant. We did not find any evidence of an association between the *GSTM1* genotypes and lung cancer risk.

Finally, the frequency of the *GSTP1 Val* allele was 0.338 in the cases and 0.349 in the controls. The frequency of the *Val/Val* genotype was slightly higher in the cases (12.3%) than in the controls (11.7%). When we analysed the association between the *GSTP1* genotypes and lung cancer risk, we found no association between individuals with the variant genotype *Val/Val* or the carriers of variant allele *Val* (Ile/Val + Val/Val) and the risk of developing lung cancer (adjusted OR = 0.83; 95% CI = 0.57-1.19 and adjusted OR = 0.83; 95% CI = 0.66-1.05, respectively) (Table 2).

### Individual effects of carcinogen metabolism genes on histological lung cancer subtype

The stratified analysis by histological type of the *CYP1A1 Msp*I T6235C, *GSTM1* present/null, *GSTT1* present/null, *GSTP1* Ile105Val polymorphisms did not reveal any statistically significant association (Table 3).

**Table 3:** **Analysis of polymorphisms stratifiedby histological type**

	**Squamous cell carcinoma**				**Adenocarcinoma**				**Small cell carcinoma**			
	**Ca/Co**	**p**^**a**^	**Unadj. OR**	**Adj.**^**b**^**OR**	**Ca/Co**	**p**^**a**^	**Unadj. OR**	**Adj.**^**b**^**OR**	**Ca/Co**	**p**^**a**^	**Unadj. OR**	**Adj.**^**b**^**OR**
			**[95% CI]**	**[95% CI]**			**[95% CI]**	**[95% CI]**			**[95% CI]**	**[95% CI]**
***CYP1A1****Msp*I T6235C												
T/T	238/247	0.384	Reference	Reference	194/193	0.909	Reference	Reference	96/108	0.070	Reference	Reference
T/C + C/C	73/64		1.18	1.38	47/48		0.97	0.83	34/22		1.74	1.45
			[0.81-1.73]	[0.86-2.20]			[0.62-1.53]	[0.50-1.35]			[0.95-3.19]	[0.74-2.85]
***GSTM*1** present/null												
present/present	160/142	0.147	Reference	Reference	109/113	0.712	Reference	Reference	56/59	0.706	Reference	Reference
> = 1 null allele	149/167		1.00	0.91	126/122		1.07	0.96	72/69		1.10	1.18
			[0.58-1.09]	[0.63-1.33]			[0.75-1.54]	[0.65-1.44]			[0.67-1.80]	[0.67-2.09]
***GSTT*1** present/null												
present/present	243/239	0.698	Reference	Reference	193/186	0.414	Reference	Reference	103/103	1.000	Reference	Reference
> = 1 null allele	66/70		0.93	0.83	42/49		0.83 [0.52-1.31]	0.76 [0.46-1.26]	25/25		1.00	1.18
			[0.63-1.36]	[0.53-1.30]							[0.54-1.86]	[0.58-2.40]
***GSTP*1** Ile105Val												
Ile/Ile	132/143	0.374	Reference	Reference	115/102	0.235	Reference	Reference	59/44	0.057	Reference	Reference
Ile/Val+Val/Val	179/168		1.15	1.12	127/140		0.80	0.69	71/86		0.62	0.61
			[0.84-1.59]	[0.76-1.63]			[0.56-1.15]	[0.46-1.02]			[0.37-1.02]	[0.34-1.08]

^a^ Chi-squared p-value.

^b^ Adjusted by age, pack-years (non-smoker, <37PY, ≥37PY), family history of any cancer, and worker in list A occupation^*^.

^*^ List A includes occupations known to be associated with lung cancer.

### Gene-environment and gene-gene interactions

An analysis of the interaction of each variant carcinogen metabolism gene alone and tobacco consumption in lung cancer risk showed that there is no gene-environment interaction (Table 4). In addition, no association was found in the analysis of the interaction between *GSTM1* present/null, *GSTT1* present/null and *GSTP1* Ile105Val polymorphisms and occupation in lung cancer risk (each gene analysed separately with occupation). However, the case of the C-allele variant in the *CYP1A1* gene could represent a possible interaction with occupation (adjusted OR [95%CI]: 2.20 [1.11-4.35] for workers in occupations included in list A), as shown in Table 5.

**Table 4:** **Analysis of interaction betweenpolymorphisms and tobacco consumptionin lung cancer risk**

	**Cases/Controls n(%)/n(%)**	**p**^**a**^	**Unadjusted**	**Adjusted**^**b**^	**p-interaction**
			**OR [95% CI]**	**OR [95% CI]**	
CYP1A1 * PY					
T/T–Non-smokers	40(5.1)/182(23.1)	<0.001	Reference	Reference	0.071
T/C+C/C–Non-smokers	11(1.4)/40(5.1)		1.25 [0.59-2.65]	1.16 [0.59-2.52]	
T/T–Smokers<37PY	139(17.6)/262(33.2)		2.41 [1.61-3.62]	2.47 [1.64-3.71]	
T/C+C/C–Smokers<37PY	32(4.1)/77(9.8)		1.89 [1.10-3.25]	1.83 [1.06-3.17]	
T/T–Smokers>=37PY	429(54.4)/187(23.7)		10.44 [6.78-16.07]	10.14 [6.46-14.99]	
T/C+C/C–Smokers>=37PY	138(17.5)/40(5.1)		15.70 [8.52-28.94]	15.38 [9.32-25.38]	
*GSTM*1 * PY					
present/present–Non-smokers	26(3.3)/102(13.2)	<0.001	Reference	Reference	0.544
>=1 null allele–Non-smokers	25(3.2)/119(15.3)		0.82 [0.45-1.52]	0.82 [0.44-1.52]	
present/present–Smokers<37PY	70(9.0)/149(19.2)		1.84 [1.09-3.10]	1.87 [1.10-3.17]	
>=1 null allele–Smokers<37PY	96(12.4)/182(23.5)		2.07 [1.25-3.42]	2.10 [1.26-3.51]	
present/present–Smokers>=37PY	279(35.9)/106(13.7)		10.33 [5.98-17.84]	10.27 [6.24-16.84]	
>=1 null allele–Smokers>=37PY	280(36.0)/117(15.1)		9.39 [5.49-16.05]	9.00 [5.49-14.73]	
*GSTT*1 * PY					
present/present–Non-smokers	40(5.1)/185(23.9)	<0.001	Reference	Reference	0.060
>=1 null allele–Non-smokers	11(1.4)/36(4.6)		1.41 [0.66-3.02]	1.44 [0.67-3.09]	
present/present–Smokers< 37PY	129(16.6)/264(34.1)		2.26 [1.50-3.40]	2.31 [1.53-3.50]	
>=1 null allele–Smokers< 37PY	37(4.8)/67(8.6)		2.55 [1.49-4.38]	2.62 [1.52-4.50]	
present/present–Smokers>=37PY	449(57.9)/161(20.8)		12.90 [8.25-20.17]	12.72 [8.55-18.91]	
>=1 null allele–Smokers>=37PY	110(14.2)/62(8.0)		8.21 [4.85-13.87]	8.05 [5.02-12.91]	
*GSTP*1 * PY					
Ile/Ile – Non-smokers	22(2.8)/93(11.8)	<0.001	Reference	Reference	0.344
Ile/Val+Val/Val – Non-smokers	29(3.7)/127(16.1)		0.97 [0.52-1.79]	0.95 [0.51-1.78]	
Ile/Ile–Smokers< 37PY	84(10.7)/141(17.9)		2.52 [1.46-4.36]	2.69 [1.55-4.66]	
Ile/Val+Val/Val–Smokers<37PY	87(11.0)/199(25.3)		1.85 [1.08-3.15]	1.79 [1.04-3.08]	
Ile/Ile–Smokers > =37PY	246(31.2)/95(12.1)		10.95 [6.06-19.76]	10.64 [6.24-18.15]	
Ile/Val+Val/Val–Smokers>=37PY	320(40.6)/132(16.8)		10.25 [5.85-19.95]	10.05 [5.98-16.90]	

^a^ Chi-squared p-value.

^b^ Adjusted by age, family history of any cancer, and worker in list A occupation^*^.

^*^ List A includes occupations known to be associated with lung cancer.

**Table 5:** **Analysis of interaction betweenpolymorphisms and worker inlist A occupation inlung cancer risk**

	**Cases/Controls n(%)/n(%)**	**p**^**a**^	**Unadjusted**	**Adjusted**^**b**^	**p-interaction**
			**OR [95% CI]**	**OR [95% CI]**	
CYP1A1 * List A^c^					
T/T–No list A	494(62.6)/544(68.9)	0.006	Reference	Reference	0.128
T/C+C/C–No list A	147(18.6)/141(17.9)		1.15 [0.88-1.49]	1.04 [0.77-1.39]	
T/T–List A	114(14.4)/88(11.1)		1.43 [1.05-1.93]	1.15 [0.82-1.62]	
T/C+C/C–List A	34(4.3)/16(2.0)		2.34 [2.27-4.30]	2.20 [1.11-4.35]	
*GSTM*1 * List A					
present/present–No list A	308(39.7)/317(40.8)	0.015	Reference	Reference	0.700
>=1 null allele–No list A	322(41.5)/357(46.0)		0.93 [0.75-1.15]	0.97 [0.75-1.24]	
present/present–List A	67(8.6)/41(5.3)		1.68 [1.10-2.56]	1.38 [0.86-2.22]	
>=1 null allele–List A	79(10.2)/61(7.9)		1.33 [0.92-1.93]	1.18 [0.78-1.79]	
*GSTT*1 * List A					
present/present–No list A	501(64.6)/525(67.6)	0.015	Reference	Reference	0.439
>=1 null allele–No list A	129(16.6)/149(19.2)		0.91 [0.70-1.18]	0.81 [0.60-1.09]	
present/present–List A	117(15.1)/86(11.1)		1.43 [1.05-1.93]	1.21 [0.86-1.70]	
>=1 null allele–List A	29(3.7)/16(2.1)		1.90 [1.02-3.55]	1.33 [0.67-2.64]	
*GSTP*1 * List A					
Ile/Ile–No list A	289(36.7)/289(36.7)	0.010	Reference	Reference	0.815
Ile/Val+Val/Val–No list A	350(44.4)/396(50.2)		0.88 [0.71-1.10]	0.84 [0.66-1.08]	
Ile/Ile–List A	63(8.0)/41(5.2)		1.54 [1.00-2.36]	1.38 [0.85-2.22]	
Ile/Val+Val/Val–List A	86(10.9)/62(7.9)		1.39 [0.96-2.00]	1.08 [0.72-1.62]	

^a^ Chi-squared p-value.

^b^ Adjusted by age, family history of any cancer, and pack-years (non-smoker, <37PY, ≥37PY).

^c^ List A includes occupations known to be associated with lung cancer.

None of the 6 possible paired combinations for the *CYP1A1*, *GSTM1*, *GSTT1*, and *GSTP1* polymorphisms showed a gene-gene interaction (Table 6).

**Table 6:** **Analysis of gene-gene interactionsof *CYP1A1 *MspI, *GSTM1 *present/null, *GSTT1 *present/null, and *GSTP1 *Ile105Val polymorphisms in lungcancer risk**

**N° of at-risk alleles**	**Cases n**	**Controls**	**Unadjusted**	**Adjusted**^**a**^**OR**	**p-interaction**
	**(%)**	**n (%)**	**OR [95% CI]**	**[95% CI]**	
**CYP1A1-GSTM1**					
**T/T-present/present**	283 (36.7)	286 (37.0)	Reference	Reference	0.923
**T/T->=1 null allele**	90 (11.7)	70 (9.1)	1.30 [0.91-1.85]	1.18 [0.80-1.76]	
**T/C+C/C-present/present**	308 (40.0)	332 (43.0)	0.94 [0.75-1.18]	0.95 [0.74-1.23]	
**T/C+C/C->=1 null allele**	89 (11.6)	84 (10.9)	1.07 [0.76-1.51]	1.07 [0.73-1.59]	
**CYP1A1-GSTT1**					
**T/T-present/present**	461 (59.9)	490 (63.5)	Reference	Reference	0.185
**T/T->=1 null allele**	152 (19.7)	120 (15.5)	1.35 [1.03-1.77]	1.26 [0.92-1.72]	
**T/C+C/C-present/present**	130 (16.9)	128 (16.6)	1.08 [0.82-1.42]	0.95 [0.69-1.29]	
**T/C+C/C->=1 null allele**	27 (3.5)	34 (4.4)	0.84 [0.50-1.42]	0.77 [0.43-1.37]	
**CYP1A1-GSTP1**					
**T/T-Ile/Ile**	277 (35.3)	266 (33.8)	Reference	Reference	0.916
**T/T-Ile/Val+Val/Val**	75 (9.5)	64 (8.1)	1.16 [0.80-1.70]	1.14 [0.74-1.75]	
**T/C+C/C-Ile/Ile**	327 (41.7)	363 (46.1)	0.86 [0.68-1.08]	0.83 [0.64-1.07]	
**T/C+C/C-Ile/Val+Val/Val**	106 (13.5)	95 (12.1)	1.08 [0.78-1.50]	0.93 [0.64-1.34]	
**GSTM1-GSTT1**					
**present/present-present/present**	304 (39.2)	276 (35.6)	Reference	Reference	0.041
**present/present->=1 null allele**	71 (9.1)	82 (10.6)	0.79 [0.55-1.12]	0.65 [0.43-0.97]	
**>=1 null allele-present/present**	314 (40.5)	335 (43.2)	0.85 [0.68-1.07]	0.85 [0.66-1.10]	
**>=1 null allele->=1 nullallele**	87 (11.2)	83 (10.7)	0.95 [0.68-1.34]	0.92 [0.62-1.35]	
**GSTM1-GSTP1**					
**present/present- Ile/Ile**	161 (20.8)	146 (19.0)	Reference	Reference	0.493
**present/present- Ile/Val+Val/Val**	212 (27.4)	209 (27.1)	0.92 [0.69-1.24]	0.88 [0.63-1.23]	
**>=1 null allele- Ile/Ile**	184 (23.8)	175 (22.7)	0.95 [0.70-1.29]	1.01 [0.71-1.42]	
**>=1 null allele- Ile/Val+Val/Val**	216 (27.9)	240 (31.2)	0.81 [0.61-1.09]	0.79 [0.57-1.10]	
**GSTT1-GSTP1**					
**present/present- Ile/Ile**	276 (35.7)	256 (33.2)	Reference	Reference	0.880
**present/present- Ile/Val+Val/Val**	341 (44.1)	352 (45.7)	0.90 [0.72-1.13]	0.81 [0.63-1.05]	
**>=1 null allele- Ile/Ile**	69 (8.9)	65 (8.4)	0.98 [0.67-1.44]	0.79 [0.51-1.21]	
**>=1 null allele- Ile/Val+Val/Val**	87 (11.2)	97 (12.6)	0.83 [0.59-1.16]	0.72 [0.49-1.05]	

^a^ Adjusted by age, pack-years (non-smoker, <37PY, ≥37PY), family history of any cancer, and worker in list A occupation^*^.

^*^ List A includes occupations known to be associated with lung cancer.

## Discussion

In this study, we have examined whether individual or joint modifying effects among four polymorphic metabolic genes were implicated in the development of lung cancer in a Caucasian population from Asturias, Northern Spain. Our results suggest that the polymorphisms *CYP1A1 Msp*I T6235C, *GSTM1* present/null, *GSTT1* present/null and *GSTP1* Ile105Val are not associated with lung cancer risk or cancer subtype.

The analysis performed in the present study between the polymorphisms studied and tobacco consumption did not reveal any gene-environmental interaction. The results showed higher lung cancer risk with higher tobacco consumption. Finally, no association was observed in the analysis of interaction between the polymorphisms studied and occupation.

Our study has several strengths, including high participation levels of eligible cases from a homogeneous population of similar ancestry and all of our control subjects being under Hardy-Weinberg equilibrium. In addition, all of our cases were pathologically confirmed. We also applied a strong quality control from genotyping (explained in detail in Methods section). Inevitably, the use of hospital-based controls is a potential limitation. The hospitals from which the cases were recruited were reference centers for all patients requiring hospitalization. Our controls were referred to these hospitals due to the presence of acute health conditions that were unrelated to lung cancer risk factors. There is always a chance of recall bias consisting of a systematic error due to differences in memories of cigarette smoking habits or occupational exposures between cases and controls. Structured interviews, like those used in this study, help to minimize this type of risk. Moreover, the prevalence of tobacco smoking and occupational exposure was in agreement with the literature. Our sample size is not large enough to find conclusive results in interaction analysis. Other genes that could participate in xenobiotic metabolism were not considered on the current study, which is another possible limitation. Therefore, our future objective is to validate these results with more individuals and powerful genotyping techniques.

Several studies have shown that the *CYP1A1 Msp*I T6235C polymorphism is associated with an increased lung cancer risk in Asian populations, especially in relation to tobacco smoking [11,32]. However, previous research, including a review of 20 studies [9] and two pooled analyses [32,53], in addition to our results suggest that there is not an established association between this polymorphism and increased lung cancer risk in Caucasian populations.

Although biological studies have shown evidence of variant genotypes in the *GST* genes, including *GSTM1*, *GSTT1* and *GSTP1*, resulting in reduced enzymatic activity in the cell, epidemiological studies do not support these findings. Many studies, including several meta-analyses and pooled analyses, support our finding that these three polymorphisms are not associated with lung cancer risk [17-19,24-27].

A large meta-analysis conducted in 2006, including 19,729 cases and 25,931 controls from 117 studies [19], found an increased lung cancer risk associated with the *GSTM1* present/null polymorphism. However, when only studies with more than 500 case/control pairs were considered, no association was observed. Similarly, pooled analyses with either non-smokers from 23 studies [53] on cases from a Caucasian population younger than 60 years old with non-small cell lung cancer [4] were not significantly related to lung cancer or disease progression.

In relation to the *GSTT1* present/null polymorphism, two meta-analyses and three pooled analyses have been performed to date. Similarly to the *GSTM1* present/null polymorphism, the meta-analysis carried out by Ye et al. [19], including 9,636 cases and 12,322 controls from 44 studies, revealed an increased lung cancer risk associated with the variant genotype of *GSTT1*. However, when only studies with more than 500 case/control pairs were considered, no association was observed. In addition, a meta-analysis of 34 studies found no association between this polymorphism and lung cancer risk in a Caucasian population [18]. The three pooled analyses, one including 34 studies [18], the second with non-smokers from 8 studies [53], and the last including cases of a Caucasian population younger than 60 years old with non-small cell lung cancer [4], showed no statistically significant associations.

Finally, a recent meta-analysis including 8,322 cases and 8,844 controls from 27 studies found no association between the *GSTP1* Ile105Val polymorphism and lung cancer risk [25] among all study participants or stratified by race/ethnicity. These findings corroborate findings from another meta-analysis of 25 studies with 6,221 cases and 7,602 controls [19] and with a pooled analysis including cases of a Caucasian population younger than 60 years old with non-small cell lung cancer [4].

Analyses of gene-gene interactions are especially important in the glutathione metabolic pathway where multiple enzymes with overlapping functions and shared substrates have been associated with susceptibility to carcinogens and toxic agents. In this study, no association was found probably due to the failure to consider an exhaustive chart of carcinogen metabolism related genes. However, other studies have found positive results in the gene-gene interaction analysis [24,27,54], which could support the notion that genome-based lung cancer risk is likely to be influenced by combinations of single risk genes of modest effect as well as synergistic gene-gene interactions.

Although it is well established that occupational exposure is an important risk factor for lung cancer [2] and the metabolic genes studied here are implicated in the metabolism of important occupational carcinogens [6,12,13], very few studies on genetic variants in these metabolic genes have been able to take occupation into account because of the difficulty to compile that information. Thus, while several studies have analysed the effect of these polymorphisms on the individual susceptibility to different cancers, particularly bladder cancer, while controlling for occupation [55-58], only five studies to date have controlled by occupational exposure in lung cancer [5,29,59-61]. Nazar-Stewart et al. [59] evaluated the occupational exposure to arsenic, asbestos, and welding or diesel products as potential effect modifiers for the *GSTM1* present/null, *GSTT1* present/null, and *GSTP1* Ile105Val polymorphisms but found no association. Jourenkova-Mironova et al. [29], Reszka et al. [5], and Risch et al. [60] used occupational exposure as a confounding variable and Yin et al. [61] used occupation as matching variable. No study has used occupational exposure; therefore, we have added to this discussion by evaluating the possible modification of the relationship between workers in high occupational risk and lung cancer development.

## Conclusions

In summary, our results suggest that the four genetic polymorphisms studied in the *CYP1A1*, *GSTM1*, *GSTT1* and *GSTP1* metabolic genes are not associated with lung cancer risk in our total population of Caucasians from Northern Spain. Furthermore, the negative results in the gene-gene interactions analysis seem to indicate that these interactions do not have an association with lung cancer development. Well-designed and powerful epidemiological studies are necessary to determinate the true role of genetic susceptibility in lung cancer.

## Competing interests

The authors declare that they have no competing interests.

## Authors' contributions

MFLC carried out the molecular genetic studies and drafted the manuscript. SMAA revised the manuscript. TP participated in the patient enrolment. AFS performed the statistical analysis and revised the manuscript. AT conceived the study, participated in its design and coordination, and revised the manuscript. All authors read and approved the final manuscript.

## Pre-publication history

The pre-publication history for this paper can be accessed here:

http://www.biomedcentral.com/1471-2407/12/433/prepub
